# Scaffolding informed consent

**DOI:** 10.1136/jme-2024-110105

**Published:** 2024-12-20

**Authors:** Dominic Wilkinson, Neil Levy

**Affiliations:** 1 Uehiro Oxford Institute, University of Oxford, Oxford, UK; 2 John Radcliffe Hospital, Oxford, UK; 3 Murdoch Children’s Research Institute, Melbourne, Victoria, Australia; 4 Centre for Biomedical Ethics, National University of Singapore Yong Loo Lin School of Medicine, Singapore; 5 Department of Paediatrics, School of Clinical Sciences, Faculty of Medicine Nursing and Health Sciences, Monash University, Melbourne, Victoria, Australia; 6 Faculty of Philosophy, Oxford University, Oxford, UK; 7 Department of Philosophy, Macquarie University, Sydney, New South Wales, Australia

**Keywords:** Personal Autonomy, Informed Consent, Philosophy

## Abstract

The principle of respecting patient autonomy underpins the concept and practice of informed consent. Yet current approaches to consent often ignore the ways in which the exercise of autonomy is deeply epistemically dependent.

In this paper, we draw on philosophical descriptions of autonomy ‘scaffolding’ and apply them to informed consent in medicine. We examine how this relates to other models of the doctor–patient relationship and other theories (eg, the notion of relational autonomy). A focus on scaffolding autonomy reframes the justification for existing ways of supporting decisions. In other cases, it suggests a need to rethink how, when and where professionals obtain consent. It may highlight the benefit of technology for supporting decisions.

Finally, we consider the implications for some high-stakes decisions where autonomy is thought to be critical, for example, termination of pregnancy. We argue that such decisions should not be free from all sources of influence—rather they should be protected from undesired influence.

## Introduction

 Autonomy is a central concept in medical ethics. It links self-governance with rationality and expresses the importance of individuals having the capacity and the freedom to make decisions about their own lives.^1–4^ A key implication is that patients decide for themselves whether or not to receive medical treatment. This manifests in the norms that relate to informed consent.^5^ When a patient requires a medical procedure or treatment, they must be provided with all the unbiased information they need in a form that they can understand. They should be given the opportunity to reflect and make a free and independent decision on whether or not to proceed. Once they have decided and communicated their decision, this must be respected. The value of patient autonomy is often given as justification for the importance of obtaining informed consent. Moreover, the process of obtaining informed consent is how health professionals respect autonomy, and how patients exercise their autonomous agency.

Patient autonomy is often described in contrast to paternalistic decisions—where the doctor makes decisions based on what she/he thinks would be best for the patient. It is commonly framed in a negative way—the absence of interferences or undue influences on decisions. Autonomy is thought to be particularly strong when expressed in terms of the absolute right of an adult with capacity to refuse any and all medical treatment that they do not wish to receive.

However, there are numerous existing criticisms of the traditional bioethical notion of autonomy.^6 7^ For example, this model is seen as too individualistic—ignoring the way in which personal and familial relationships and wider groups are viewed (by at least some) as vital to decisions. The model also neglects the numerous ways in which our choices are shaped and directed by external influences, in ways both subtle and profound.

In response to these criticisms, some have suggested that we should reduce the emphasis placed on autonomy. For example, given the flawed nature of individual decision-making (susceptibility to bias or other forms of irrationality), perhaps medical paternalism is not as bad as it has been taken to be and is sometimes justified.^8^ Along the same lines, some forms of influence on decision-making that are taken to bypass rational processes (eg, nudging) may not be as great a threat. Alternatively, we might choose to broaden the scope of autonomy to encompass more than the individual—allowing and embracing the possibility of patient decisions being socially embedded or relational, made by or in concert with family members, partners or the community.

In this paper, we will suggest a different, although complementary, approach. We will argue that the emphasis, in the traditional concept, on independence and freedom from influence leads to decision-making that is unhelpfully separated from the epistemic structures that support our choices. We need those support structures to make autonomous decisions, we will argue. Our everyday decision-making is interdependent, not independent. We first describe and defend the philosophical notion of ‘scaffolded autonomy’. Once we recognise the scaffolds that are necessary for patients to decide, it will become clear that respecting patient autonomy imposes a positive duty to provide the necessary support structures. It may require accepting or even inserting (rather than removing) influences on patient decisions.

## Scaffolded autonomy: theoretical background

How precisely is autonomy scaffolded? All but the most caricatural individualists recognise that good decision-making often requires support of various kinds. In much lower stakes contexts than in medicine, people often want support in their decision-making—a person buying a car might want the advice of friends, or to talk it over with family. Unsurprisingly, they might reasonably want support from others in making decisions about treatment. Additionally, medical decision-making typically has implications for people beyond the person whose treatment it is. For the duration of treatment and convalescence, a man might be unable to contribute to caring for his children, for example, and he might want to weigh that fact in his decision-making. Another person might think that it would be burdensome for her partner to see her suffer for a prolonged period and prefer to forgo treatment on that basis. Insofar as these decisions take into account other people and make assessments of how treatment, or non-treatment, might affect them, they are reasonably taken in consultation with others.

In what we might call a socially supported model of decision-making, individuals make their decisions with input from others. They seek opinions and advice from family and friends, and information from medical professionals, and then attempt to weigh that information in coming to a decision that reflects their own values and outlook. Such socially supported decisions are very plausibly better for the input of others: a broader range of considerations are brought to bear than the individual could marshal on their own. But the final decision-making reflects cognition that is fully the individual’s own. Properly scaffolded models, however, go beyond socially supported models. On the latter, decision-making reflects cognition that is distributed across agents and across the environment. Scaffolded autonomy draws its inspiration from distributed models of cognition.^9 10^ In these models,other people and their opinionsare not merely inputs into decision-making, they play a role in constituting it.

Distributed models of cognition are counterintuitive, but there is a case for saying that agents engage in such cognition in everyday life. This fact has been obscured in the literature, where the paradigm cases of distributed cognition have involved formal procedures for distributing tasks. For example, Hutchins’^11^ influential argument for the conclusion that the navigational knowledge of a large ship is distributed across multiple agents and even artefacts turns on how tasks are distributed across different agents none of whom are able to integrate the information or to deploy it on their own. It is because tasks are assigned via procedures settled in advance that the vessel safely docks.

But distributed cognition can be emergent, without the need for formal mechanisms. Cultural evolutionary thought emphasises how human beings have been distributing cognitive tasks across groups for millennia, to such a degree that we may have psychological adaptations that enable it. For example, the prestige bias^12^ prompts us to copy the behaviours of successful individuals, thereby saving us the effort of trial and error learning, while the conformity bias^13^ allows us to adopt ways of behaving likely to be suited to the local environment. Regardless of the mechanisms, agents do seem flexibly and intelligently to outsource many of their own cognitive tasks, in a complex dance of relying on and thinking with others.

Distributed cognition can involve relying on others to perform certain tasks so that we don’t have to. A simple illustration is that of transactive memory^14^ in which individuals rely on one another to remember facts and events. For example, an implicit division of recollective labour might develop in a couple, with one partner specialising in remembering birthdays and the other bills, or one in remembering what films they have seen and the other in which restaurants they have tried. This sort of arrangement typically evolves over time, without planning.

But distributed cognition is never entirely passive. Even in cases in which we rely on one another, we potentially play an active role; for example, couples in which a division of cognitive labour emerges may nevertheless depend on one another to prompt them appropriately, for example, by recalling the context in which they saw the film or the occasion on which they ate at the restaurant. They are not mere storehouses of knowledge for one another, but active participants in evoking one another’s memories.

The distribution of knowledge in everyday life also involves depending on anonymous others. We depend on others to know things (so we do not have to) by virtue of their roles. Commuters depend on bus drivers to know the route to downtown, while drivers depend on those who laid out the roads to know the most efficient route between two cities. Even in cases like this, where we do not have an ongoing relationship and may not be in a position to enter into dialogue with the person we rely on, we are not purely passive. Rather, we exercise epistemic vigilance: a pervasive, if typically implicit, sensitivity to evidence of the reliability of the person and their assertions. We are sensitive to evidence of their competence, of their attitude towards us and those we care about, the degree to which their testimony reflects a consensus, and of course to its plausibility.^15–17^

Our epistemic dependence is so deep and so automatic, we may be entirely unaware we engage in it. In a well-known series of experiments, Frank Keil and colleagues showed we suffer from an illusion of explanatory depth: we think we understand mechanisms and processes until we are asked to actually explain them; at that point, we admit we do not know.^18 19^ The illusion of explanatory depth is widely understood as showing we are unjustifiably confident, but Keil himself puts forward a different explanation: we take ourselves to know how these things work because we outsource the knowledge to others.^20^ That is, in some sense, we do know how flush toilets and pianos and zippers work; it is just that the knowledge is stored in other people’s heads, not our own. Our mistake consists in thinking the knowledge is internal, rather than distributed across agents. This interpretation is bolstered by the finding that people report knowing things at a higher rate when they are told that others in their community know them, or when they have access to the internet, as opposed to circumstances in which these conditions are not satisfied.^21 22^

Importantly, for our purposes, this dependence is not restricted to the domains of facts or procedures. We may depend on others for values too. Just as a religious Catholic might assert that God is three persons in one while depending on priests and theologians to give that assertion content, or a more secular person claim that the theory of evolution is true, while outsourcing what the theory actually says to evolutionary biologists, someone might commit to the value of equality while having only a vague idea of what equality consists in. After all, ‘equality’ is a difficult and contested term; even among specialists who share a broad value orientation, there is a debate over what it requires and commits us to.^23–26^ The same holds for other values, like freedom. In understanding this value, it is notoriously contested what weight we should give to the absence of constraints (freedom from) versus capabilities (freedom to) . Ordinary people may, therefore, partially outsource the details of their own commitments to others.

Of course, deference cannot be total. If each of us deferred to others like ourselves for our own commitments, the work of making our commitments precise and adjudicating conflicts could never occur: we would each be waiting for others. Instead, we each make proposals as to how to cash out our commitments (even if only the implicit proposals that consist in manifesting a preference for one response rather than another). In practice, each of us probably defers to a very great extent with regard to most of our own commitments while being quite active in refining and defining a very small number of them. That is itself an instance of the intelligent use of the division of cognitive labour: since refining these concepts is difficult, it is rational to build up a body of expertise that’s focused narrowly. A small number of expert people working together to define a concept will be more successful than a large number, none of whom have the time to develop special expertise.

While deference is pervasive, we are typically unaware of its extent. We are epistemic individualists: we value thinking for ourselves and by ourselves. Kant famously called on us to dare to think: have the courage to use our own understanding,^27^ and his call has been taken up by many. The very fact that people withdraw their claim to know something when the illusion of explanatory depth is exposed shows that while we implicitly rely on others, our explicit theories of knowledge are individualistic. Both our pervasive dependence on others and our epistemic individualism have important implications for situations in which we are asked to make decisions that reflect and manifest our values.

My values are genuinely mine, in a non-extended way; outsourcing does not go all the way down. It may also be true that none of my values is non-negotiable; what might be non-negotiable is that any value commitment must be a natural development from my values taken as a whole. I know, without assistance or scaffolding from others, that I value equality and fairness and democracy. But I may not recognise how difficult it is to give these concepts the precision needed to apply them and to adjudicate the inevitable conflicts between my values. I am also unlikely to recognise how I actually negotiate these conflicts and make my own commitments sufficiently precise for application by deferring to others I trust. I fill out the details and the commitments by observing and engaging with others, who I take to share my values.

On scaffolded models, therefore, agents’ commitments, beliefs, values and ideals are not just ‘in the head’. Rather, they are constituted by the dispositions and inclinations of the agent—on her own*—*and by other agents she defers to. Other agents provide much of the content to our values. This is true even for those values that lie at the heart of autonomy: the autonomous individual may value self-determination (say), but they typically—and perhaps always—cannot provide a precise content to that value on their own. To understand what she herself means by self-determination, she needs recourse to others (of course, this kind of scaffolding is typically mutual: the agents she defers to provide a content for her valuing self-determination may defer to her in turn when it comes to some other commitment).

If this is true, autonomy is necessarily scaffolded via two routes. First, our very valuing of autonomy is itself scaffolded. While it is certainly possible—normal—to know that one values autonomy, assuming one does, it is rare that one can make the content of this value precise. The ‘thin’ content of autonomy, as realised by the individual herself without scaffolding, can guide many of her decisions, but when values conflict or in hard cases, she may need to guide her decision by reference to a more determinate understanding, and she will usually need scaffolds to provide this more determinate understanding. Second, though, this is true for the values that are supposed to be protected by autonomy. We value autonomy because it allows each of us to make decisions that are guided by our own values, our own conception of the good. But if values are scaffolded, then we can express our own values only with the support of scaffolds. In a slogan, if you want your decisions to express your own self-determination, it cannot be entirely self-determined—not if by ‘self’ one has in mind only the person, cut-off from the world and from others.

## From scaffolded cognition to informed consent

We greatly underestimate the contribution that others make to our own decision-making; we may even see them as interfering. Thus we think that a good decision is one that we make on our own. Our informed consent procedures largely reflect this thought: while of course all sides fully accept that patients making difficult decisions must be provided with relevant information, we aim to screen off others beyond that. We aim to allow the person to express her values and commitments, not those of others. But while her values and commitments are irreducibly her own, she relies on certain trusted others to make her own values more precise for her, in exchange and in dialogue with her. In screening off these others, we prevent her from expressing her own values.

It will be helpful to clarify how scaffolding relates to existing models of informed consent. One highly influential and much-cited account is that of Emanuel and Emanuel.^28^ In their seminal paper, they analysed the ‘doctor–patient relationship’ as it relates to information provision and decision-making around medical treatment. They described four types of interaction, including at one end of the spectrum, an ‘informative model’ (the doctor provides relevant factual information and the patient decides) and at the other end a ‘paternalistic model’ (the doctor determines what would be best and obtains the patient’s assent to providing this). In between, they described an ‘interpretive model’ with the physician, akin to a counsellor, supporting the patient to elicit and apply their values to the decision. Finally, they proposed and recommended a ‘deliberative model’, with the doctor engaging in debate and seeking to persuade the patient of a course that would best promote his or her interests. In the decades since others have expanded and developed this taxonomy. For example, Veatch^29^ proposed deep value pairing (wherein patients seek out physicians with whom they share relevant values); in response, Savulescu defended a ‘liberal rationalist model’ (similar to the deliberative approach) based on promoting objective well-being.^30^ More recently, Davies and Parker have articulated an ‘appointed fiduciary’ model, providing a framework for situations where patients explicitly choose for doctors to make a decision for them: ‘doctor, you do what you think is best’.^31^

To some degree, the strengths and weaknesses of these different models can be illuminated by reference to scaffolded autonomy. For example, the two extremes identified by Emanuel and Emanuel (informative and paternalistic) conflict with the importance of scaffolded autonomy—the former because it offers no support to patient decision-making, the latter because the patient has little or no choice offered to them. The ‘appointed fiduciary’ model may provide helpful epistemic scaffolding for patients who prefer to defer to an expert (the health professional). That might be particularly applicable where the patient’s values line up with those of the physician (eg, through deep value pairing). The deliberative and interpretive models will also be helpful forms of scaffolding in some cases. One straightforward way to scaffold autonomy (not included in Emanuel’s seminal publication, though often implicitly or explicitly included in modern accounts of shared decision-making) is for professionals to engage with patients in a meta-decision-making conversation—we return to that below.

However, a significant limitation of these doctor–patient models is that they all focus on a limited interaction in the consulting room between the patient (possibly with partner) and the professional. This potentially ignores the ways in which others outside the consulting room may play important roles for the patient in considering and eliciting the relevant values and applying them to the medical decision at hand.

Other models of informed consent resemble scaffolded autonomy more closely. In a familial model, patients make decisions in consultation with family members.^32^ On relational models, informed consent occurs when the patient is in an appropriate relation to an autonomy-supporting background^33 34^; in practice, relational models tend to be dialogical. The scaffolded approach overlaps with these models but differs from both in its emphasis on the way in which others play an epistemic role in our thought.^i^

Relational models of autonomy are extremely well developed in philosophy. Such approaches are motivated by a dissatisfaction with the individualism of notions of autonomy that used to dominate ethics; with what has been called the Marlboro Man model,^35^ on which the autonomous individual is alone, self-sufficient, figure. Feminist philosophers instead emphasised how even the Marlboro Man was dependent on others, at least developmentally. Relational theorists often go further, arguing that our dependence on others is not merely causally necessary for us to be autonomous, it may also be constitutively necessary. We are social beings, and even when an individual is taken from their social context, they continue to use concepts and understand themselves in a way that involves an implicit dialogue with others.^36^

Relational models stress how the psychological dispositions and the understandings required for autonomy are dependent on our embeddedness in social relations. Such models differ among themselves. Some are substantive accounts: on these views, autonomy requires that the agent endorse certain substantive values. Others are proceduralist, holding that any value at all might be autonomously held, so long as the agent is prepared reflectively to endorse it.^37^ Some mix substantive and procedural requirements, holding that while autonomy depends on reflective endorsement, certain values entail a self-effacement incompatible with such endorsement.^38^

The scaffolded model is compatible with every version of relational models, but in certain respects goes beyond them. Relational models stress that social scripts^39^—that is, certain ways of behaving expected of those who occupy social roles—stereotypes and expectations may enhance or undermine autonomy. The woman who is socialised into subordinating herself to others may find it harder to be autonomous; conversely, the woman who has had her self-trust bolstered is at an advantage. Scaffolded models endorse these claims, but go beyond them in claiming that our concepts and values are always and necessarily cashed out in dialogue with others. Scaffolded models are closer to constitutive versions of relational autonomy,^40^ in that they stress that such elaboration is an ongoing project (we never refine our concepts in a way that will always allow us to act appropriately in response to novel situations; rather, they develop over time in response to changing circumstances). However, whereas constitutive versions of relational autonomy require that external conditions be apt for the agents’ psychological capacities, scaffolded models maintain that the boundary between the agents’ capacities—specifically, their epistemic capacities—and the external conditions is at best blurry. External conditions might be said to be partially constitutive of these capacities. This claim is distinctive of the scaffolded model.

The scaffolded model might also usefully be compared with family-oriented models of informed consent. Such models are especially associated with non-Western perspectives. Some psychologists claim that the nations of Europe, North America and the Anglosphere are psychologically WEIRD: Western, Educated, Industrialised, Rich and Democratic nations have populations that are far more individualist than other nations.^41 42^ Family-oriented models emphasise the place that the individual occupies in her family. This has at least two rationales. First, her well-being is not merely a matter for her; rather, her flourishing and her suffering impact everyone in the family. Others have skin in the game. Second, her flourishing is intertwined with that of others; she cannot do well independently of others, and conversely, they cannot flourish without her flourishing. As a consequence, healthcare decisions are never a matter for her alone.^32 43^

The scaffolded model is compatible with—but does not presuppose—the family-oriented model. In schematic form, it takes no stand on whether our flourishing is intertwined with others. It insists, rather, that our capacity to reason well is intertwined with others, and that this is true in WEIRD and non-WEIRD cultures alike. It is committed to denying a thoroughgoing individualism, though it is agnostic whether there are nevertheless psychological differences across cultures. On scaffolded models, WEIRD people may be committed to an individualistic conception of autonomy, but they (too) are going to need to work out what that actually amounts to together with others.

There is a lively debate between some proponents of relational and family-oriented conceptions of autonomy. Some of the former think that family-oriented conceptions promote autonomy-limiting social scripts.^44^ Others take this criticism to seriously undervalue the autonomy of preferences that are, admittedly, shaped by circumstances.^45^ Again, scaffolded approaches need not take a stand on this debate. It is compatible with scaffolded approaches that deference to (certain) others undermines autonomy. Rather, it maintains that autonomy requires apt deference to, and dialogue with, those who share—and thereby help constitute—our values. While scaffolded approaches do not require that these others be committed to promoting our autonomy, it does require that they manifest sufficient respect toward us to allow us to engage with them and be heard by them. Because it is neutral on the locus of value, and on the substantive content of autonomous preferences, it is compatible with a variety of other approaches. The best-scaffolded account will draw from others. That, too, is an expectable upshot of the division of cognitive labour.

## Scaffolding autonomy in informed consent

The scaffolding metaphor may be useful for describing how we see this concept being applied. When someone embarks on significant renovations or building on their home, it is often a crucial first step to put in place necessary scaffolding. The scaffolding facilitates the process of building. But whether, or how much scaffolding is required will depend on the work needed, on the nature of the building, etc. Scaffolding is also dynamic. It may be needed at the start of work, but later be able to be removed (like training wheels on a bicycle). Some scaffolding might be required to permanently buttress a building, and cognitive scaffolds, too, might be intended to remain in place.

When physicians interact with patients, a cognitive scaffold is already in place. But that scaffolding may not be apparent. Some scaffolds are widely shared (the physical environment—eg, the layout of roads—scaffolds cognition for everyone ubiquitously). But some scaffolds are peculiar to an individual and reflect their idiosyncratic cognitive history. Some types of scaffolding are common; for example, reliance on family and friends. Some are less common, and a physician may not suspect the need for them. There is, accordingly, a risk that the informed consent procedure inadvertently screens them off. Even if patients are encouraged to consult with family, say or the family is brought into the consulting room, important scaffolds may be absent.

Physicians can and should ask the patient what scaffolding they need: that is, who they would like to talk important decisions over with. However, that may not always be effective. Since reliance on scaffolding is often implicit, we may not be well positioned to see the need for them ourselves. There is a risk our epistemic individualism might lead us to inappropriately withdraw from our usual social scaffolding. Of course, patients have a right to do that if they choose; nevertheless, they may appropriately be encouraged to reflect on how they go about making decisions and assured that for many people, social scaffolding is not merely appropriate but might be essential for genuine autonomy.

Emanuel and Emanuel’s classic paper included a clinical example to help illustrate how their four models would diverge in their approaches.^28^

*A 43 year old premenopausal woman has recently discovered a breast mass. Surgery reveals a 3.5cm ductal carcinoma with no lymph node involvement; the tumour is oestrogen receptor positive. Chest X-ray, bone scan and liver function tests reveal no evidence of metastatic disease. She has recently divorced and is working as a legal aide.*

How would the Scaffolded approach suggest that the physician approach this case? Like Emanuel and Emanuel’s physicians, she might start by outlining the relevant factual information about available options and the advantages and disadvantages of each. But before proceeding further, our imagined physician might pause:

Before we talk more about which option might be best for you, or what you might wish to do, it might be helpful to talk in general about difficult decisions. For some people it is very important to talk decisions through with other people, for example their partner or a close friend, their family or sometimes a spiritual advisor. What about you? When you’ve made big decisions in the past, is there someone with whom you’ve found it helpful to talk the decision through?

If the patient answers that they prefer to make big decisions by themselves, the physician might then ask whether they would consult someone if the decision was not especially important. If the patient describes scaffolding for smaller decisions, the physician might then encourage them to use such support for big decisions as well. They should not insist, however. The physician should appropriately provide the information that decision-making is often socially scaffolded but cannot and should not require that the patient act on that information.

Next, the physician might add

Also, different people who I talk to need different things from me to help them make decisions. For example, some people just want to know the facts and then prefer to go away and make a choice. Others find decisions very difficult and ask me to make a decision for them. Still others find it hard to know what they think. We talk together about what is most important to them, what their priorities are and what they most fear, to help work out what might suit them best. Still others who I talk to want to know what I would recommend or advise and why—though they may choose to make a different decision in the end. What about you? How can I be of most help?

Again, in the light of our tendency to epistemic individualism, patients might choose an ‘informative’ model of decision-making because that is what they (mistakenly) think it means to be fully autonomous. Here too, the physician might appropriately push back, by providing the information that many people think that such decisions do not truly express agents’ autonomous will. Ultimately, the physician should respect the patient’s decision.

Table 1 illustrates how the scaffolded model might be seen as distinct from the other classic approaches to informed consent. As noted, it differs from the other models in its conception of patient autonomy, but also in how it conceives of the physician’s role. Rather than being seen as the patient’s teacher or counsellor, in the scaffolded model, the physician is perhaps akin to a walking guide. How much assistance the other person needs will depend on the nature of the journey and the experience, confidence and preferences of the walker. In some instances, the guide will (like a sherpa) shoulder a significant load and provide invaluable and tangible support. In others, the guide may indicate on a map a course that the walker ultimately chooses not to take, preferring to follow their own path. In the table, and in the hypothetical dialogue above, the scaffolded model is also envisioned as being complementary to the other models. For example, it would be compatible with a scaffolded approach if a patient were to choose to defer to the physician’s judgement.

**Table 1:** Scaffolding autonomy, in contrast with other models of informed consent/doctor–patient relationship (modified from Emanuel and Emanuel)^28^

	Informative	Interpretive	Scaffolded	Deliberative	Paternalistic
Patient values	Defined, fixed, known	Unknown, conflicting, requiring eliciting and clarification	**May be held or shared with others** **May emerge only in interaction with others**	Potentially open to challenge and revision through discussion with the physician	Objective and known by the physician
Physician’s obligation	Providing medical factual information. Implementing patient’s choice	Value elicitation in conversation with the patient, implementing patient’s choice	**Acknowledge the importance of significant others both for understanding and values. Respect patient’s preferences for process of decision-making (and choice**)	Identifying the ideal option, discussing and debating, implementing patient’s choice	Promoting the patient’s well-being—recommending and implementing the best treatment option.
Patient’s autonomy	Manifest in patient choice and ultimate decision	Expressed in enquiry and process of value elicitation	**Requiring support of others. Distributed and shared (to a variable degree**)	Promoted by rational deliberation and making choices in the light of understanding of best reasons	Secondary to consideration of objective well-being
Physician’s role	Technical expert	Counsellor	**Sherpa or walking guide**	Friend or teacher	Guardian or parent
	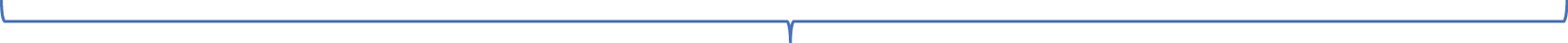 **Scaffolded support to patient’s preferred model**				

## Practical implications of scaffolded autonomy for informed consent

We have described the way that this account might reframe and reinforce existing models or ways of obtaining informed consent.

### Actively identifying support

We have already suggested that on this approach health professionals should actively enquire into the supports that patients might need to make decisions. This conclusion is not novel and is consistent with many existing guidelines. For example, the guidance to shared decision-making produced by National Institute for Health and Care Excellence encourages health professionals to ‘ask the person if they want to involve family members, friends, carers or advocates…if so, include them as a way to help the person: actively engage in the discussion; explain what matters to them; make decisions about their care; remember information they have been given…’.^46^ However, the scaffolded model goes beyond existing guidelines, not only in its rationale for such decision-making, but also in its provision of information that good decision-making is typically scaffolded.

### Explicitly pluralistic in approach

By offering and supporting the patient to choose a preferred style of decision-making, scaffolded autonomy could support a range of different ways of deciding. A scaffolded approach does not assume that, for example, a deliberative approach is the optimally autonomous way of making decisions.

### Encourage staged consent conversations

Because a patient’s autonomy is likely to be supported by others who are not in the room at the time that a physician or other health professional has their initial conversation, it may often be helpful for the patient to have a chance to discuss their options with others. Again, the idea that consent occurs over a series of conversations rather than a single encounter is not new. However, scaffolding provides a separate rationale for this.

### Scaffolding capacity

One obvious, and we think uncontroversial, place where scaffolding is needed is for patients who currently lack decision-making capacity, but who may be able to make decisions if given sufficient support. In jurisdictions like the UK, there is a legal obligation on professionals to seek and (if possible) provide the support necessary for patients to be able to make decisions for themselves.^47^ One way of doing so is to draw on the social networks around a patient (eg, family, carers), to help the individual to understand, reflect and express their preferences. Such support is a form of scaffolding. However, in the model that we have described, scaffolding autonomy goes beyond providing the minimum necessary support to cross a threshold of decision-making capacity. It applies to typical situations where patients already have the relevant ability to make decisions. At least where patients wish for this, there can be important ways for health professionals to explicitly attend to the epistemic structures that are necessary for patients to make the best decision that they can in the circumstances.

In these respects, scaffolded models are modestly different from existing models; they differ in that they recognise agents typically lack insight into their own use of scaffolding, and therefore, gently encourage its use. Beyond that, scaffolded models may differ from existing models in the following respects.

### Supporting remote and digital consent

With advances in relevant technology, particularly since the COVID-19 pandemic, there has been a surge in interest in digital consent procedures in medicine. Discussions of such processes often highlight the pragmatic advantages (eg, convenience, accessibility and efficiency), as well as potential concerns about acceptance, quality of understanding or equity.^48^ Often the hope is that remote consent will not be (too) inferior to in-person consent procedures. But scaffolding may offer a distinctive argument in favour of such forms of consent: it may make remote consent better than the traditional alternative in some cases. By allowing patients to access information within a familiar environment and context, remote consultations may—for some patients at least—give patients greater access to the scaffolding that they need to be autonomous.

The possibility of using large language models to support consent conversations may extend this further by giving patients considerably greater flexibility to source and engage with the information relevant to informed consent as well as a process of deliberation.^49 50^

### Accepting some influences on patient decisions

Because patients’ decisions and autonomy are always supported and influenced by others, some cases that have caused concern may be less threatening. For example, there has been much discussion of the ethics of nudging, with many ethicists concerned that nudges represent paternalistic interferences with autonomous decision-making.^51 52^ The recognition that decision-making is appropriately scaffolded promises to transform this debate, if and to the extent that nudges may appropriately be seen as scaffolding decision-making.

A parallel argument may defend the appropriateness of scaffolds in other cases. For example, sometimes health professionals may be concerned about situations when patients’ decisions about medical treatment appear to be significantly influenced by other family members (eg, by spouse, or parents, or children). They may worry that such palpable influences undermine patient autonomy. One way of evaluating their significance would be to ask, hypothetically, ‘if this influence were not present, would the patient make the same decision’. Where the answer is ‘no’, there could be a strong sense that the patient’s decision is ipso facto non-autonomous. However, on the scaffolded approach, the truth of such a counterfactual does not necessarily mean that a patient’s decision is non-autonomous. Our decisions are always (or virtually always) guided by others, and such guidance will sometimes change the decisions we make. To determine whether guidance or scaffolding is problematic, we need to consider not merely its magnitude, but its nature and meaning within the patient’s life. We suggest that the scaffolding approach should transform our understanding of ‘influences’: if input or guidance from others is partially constitutive of our decision-making, they are not best seen as influences on it at all. Rather, they are scaffolds of it.

## Objection: autonomy-critical decisions

Some might accept the appropriateness of epistemic deference and scaffolding autonomy for decisions outside medicine. They may even feel that decisions about surgery or cancer treatment could be appropriately scaffolded. However, they could express concern that in certain medical contexts it is particularly important that decisions are the patient’s own—that they are not influenced or made on the patient’s behalf by others (even if the patient has chosen that). These are cases that turn centrally on deep personal values, and for which (on some views at least), there is no right or wrong decision, either morally or prudentially. Examples of what we might call ‘autonomy-critical decisions’ include decisions to terminate a pregnancy, to request assisted dying, or to donate a solid organ (eg, a kidney). In prenatal testing, this is expressed in the common suggestion that counselling should be ‘non-directive’: clinicians should be neutral about options of continuing or terminating a pregnancy.^53^ For debates about assisted dying, there is often particular concern about pressure or influence on decisions. ‘if a person is motivated by means other than his own will, for example, through external coercion, then patient autonomy is infringed’.^54^ For organ donation, some countries require independent parties to interview prospective donors to ensure that they are not under any duress or coercion to donate.^55^

If respecting patient autonomy requires the absence of influence in such cases, does that undermine the argument in favour of scaffolding autonomy?

One response might be to accept that these decisions are qualitatively different from others in medicine. Since they turn only (?) on personal values that are introspectively knowable to the individual, there is no epistemic need for external scaffolds, and no possibility of using others to obtain information or advice. However, the scaffolded model entails scepticism about this response. It insists that though a patient has every right to the final say, how their values are realised depends on their scaffolding. Making our values precise is something we do in concert with others (including the health professional). A decision one person makes on her own may not appropriately express her values, precisely because she made it on her own. Scaffolding, if it applies to any value based decisions, might also apply to these.

It remains true, however, that some influences are inappropriate. Though we rely on others to apply our own values, it is also a familiar fact that we can be pressured or coerced into acting on values that are not our own. What distinguishes these decisions from others in medicine is not that autonomy is more important, nor that these are quintessentially decisions best made by the person on their own. Rather there are particular concerns in pregnancy termination, for example, regarding the dangers of certain kinds of coercive influence. It may be that for this type of decision a heuristic of shielding patients from external influences might be appropriate.

An ideal informed consent procedure would support desirable scaffolding of decisions while screening off inappropriate influences. We acknowledge that such screening will be challenging. One patient might engage in dialogue with parents or with advisors to make her values precise and to think through the possibilities, whereas for another patient these same influences might be autonomy subverting. One patient might rely on friends; another on social media. Understanding which influences scaffold deliberation for each individual requires deep acquaintance with their life and their context, and medical professionals are unlikely to have such individualised knowledge.

In the face of this sort of diversity of scaffolds, and a corresponding diversity of inappropriate influences, medical professionals might use heuristics instead. Warton *et al*’s^56^ model of ‘reproductive deliberation’ provides an approach to prenatal genetic counselling that—drawing on relational autonomy—engages in just such a scaffolded approach. On their model, genetic counsellors draw on their skills to assess what information a person needs to make a decision and to explore their values with them, while being ready to step back completely at the patient’s request. They fully recognise ‘the importance of interdependence and social context’ for making decisions (581), through counsellor involvement in the process of decision-making and with the person free to involve persons outside the healthcare setting. Our scaffolding model draws inspiration from theirs, but goes further. As imagined in our dialogue (above) with the woman with breast cancer, on our model health professionals involved in the informed consent process must be sensitive to whether the person would benefit from the involvement of trusted others and encourage them to seek such involvement, rather than merely leaving them free to involve such partners. Of course, the patient must be entirely free to resist such encouragement. The doctor or counsellor might explicitly discuss the scaffolding approach, to highlight how such guidance is essential to good decision-making for many people and to indicate the range of scaffolds others find useful, but the patient retains ultimate control over the process.

The doctor may also open discussion about unhelpful influences:

Although it is often a good idea to talk with other people about important decisions, some advice might not be helpful. Some patients I care for describe feeling pressure from their family or friends to make decisions that wouldn’t actually be right for them. Is that something that you’ve experienced, or that you’d be worried about?

We urgently need research into what approaches or heuristics counsellors or physicians might use in this context and how reliable these are. For example, how often can cultural background inform the physician (who may not share that background, of course) of which influences are inappropriate and which autonomy-supporting? Do interactions between patients and family members provide clues about whether they exercise an autonomy-supporting role in deliberation? Reliable screening off of inappropriate influences depends on much better answers to these questions.

## Conclusions

Autonomy requires that our decisions be our own and that they reflect or manifest our values. In light of these facts, ethicists have emphasised the need to limit the influence over our decisions. On many models, others should provide information, but not pressure in one direction or another. Even relational models typically emphasise relations as a background condition of good choice, rather than as an active support for it during deliberation.

The scaffolded model argues that decisions must be appropriately supported in order for them to reflect our values. We rely on others to give any precision to our values (just as they, in turn, rely on us). While influences can undermine autonomy, influences of the right sort are required by it. The scaffolded model offers a new rationale for some extant proposals. It also goes beyond them, in insisting that capacities are themselves partially constituted by other agents (and perhaps extra-agential supports of various sorts, too).

The scaffolded model faces an enormous challenge, from the cultural myth that important decisions should be made by each of us alone. Our epistemic individualism entails that medical professionals and patients themselves (and perhaps bioethicists too) will be reluctant to see the need for scaffolded procedures. They may resist them if they are proposed. A patient might think that the decision they face is too important to scaffold: they might refuse scaffolding when it is most vital.

One way to respond to such a problem is by making scaffolding routine. However, any such a proposal faces difficulties: how are medical professionals or policy-makers to distinguish the scaffolds that support autonomous decision-making from irrelevant and autonomy-undermining influences of those who do not share the patients’ values or value their well-being? Trusted friends and family members often constitute an autonomy-enhancing scaffold, but sometimes they represent an obstacle to autonomy.

The best response would be to overcome the cultural myths that support epistemic individualism, but that is obviously a long-term project. Perhaps the best we can do in the meantime is to inform patients that good decision-making is typically scaffolded and give them opportunities to draw on their usual social networks. It would be good practice in this context not to try to discuss options and reach decisions in a single encounter, but, to encourage patients to return signed informed consent forms at a later date (alternatively, or as well, they might be asked to bring a trusted friend or family member with them to a follow-up appointment to ask questions of the doctor and patient, to help them make the best decision about treatment options). In doing so, we bring it about that they thereby return to the environment in which their decision-making is scaffolded, even if they do not see things that way. We can suggest that they talk the decision over with trusted people while emphasising it is their decision. Of course, such an approach is not always practical, but it might be the best we can hope for to scaffold decision-making in the near term.

## Data Availability

No data are available.

## Footnotes

We suggest in this paper that ‘scaffolded autonomy’ represents a distinct approach to that of ‘relational autonomy’. However, given the overlap, we acknowledge that others may prefer to conceive of this as a new form of relational autonomy.
